# A ten-year study of immunogenicity and safety of the AS04-HPV-16/18 vaccine in adolescent girls aged 10-14 years

**DOI:** 10.1080/21645515.2019.1625644

**Published:** 2019-07-17

**Authors:** Tino F. Schwarz, Li-Min Huang, Alejandra Valencia, Falko Panzer, Cheng-Hsun Chiu, Annabelle Decreux, Sylviane Poncelet, Naveen Karkada, Nicolas Folschweiller, Lan Lin, Gary Dubin, Frank Struyf

**Affiliations:** a Institute of Laboratory Medicine and Vaccination Centre, Klinikum Würzburg Mitte, Standort Juliusspital, Würzburg, Germany; b Department of Pediatrics, National Taiwan University Hospital, Taipei, Taiwan; c Department of Pediatrics, Fundación de Santa Fe de Bogotá, Bogota, Colombia; d Practice for Pediatric and Adolescent Medicine, Mannheim, Germany; e Department of Pediatrics, Chang Gung Memorial Hospital, Chang Gung University College of Medicine, Taoyuan, Taiwan; f GSK, Wavre/Rixensart, Belgium; g GSK, King of Prussia, PA, USA; h Takeda Pharmaceuticals, Glattpark-Opfikon, Zurich, Switzerland

**Keywords:** HPV-16/18 AS04-adjuvanted vaccine, HPV-31, HPV-45, non-vaccine types, cross-reactivity, immunogenicity persistence, safety, predictive modeling

## Abstract

This study assessed long-term immunogenicity and safety following 3 doses of AS04-adjuvanted human papillomavirus (HPV)-16/18 L1 virus-like particle (VLP) vaccine in females 10–14 years old. Girls included in the immunogenicity subset in the primary controlled, observer-blinded, randomized study (NCT00196924) who received 3 doses were invited for a 10-year follow-up (NCT00316706 and NCT00877877). Serum antibody responses against HPV-16/18 (vaccine types) and HPV-31/45 (non-vaccine types) were measured by enzyme-linked immunosorbent assay (ELISA) using type-specific VLP as coating antigens. Serious adverse events (SAEs) and pregnancy information were recorded. At Month (M) 120, all subjects (N = 418, according-to-protocol immunogenicity cohort) were seropositive for anti-HPV-16/18 antibodies. Geometric mean titers (GMTs) were 1589.9 ELISA Units [EU]/mL (95% confidence interval [CI]: 1459.8–1731.6) for anti-HPV-16 and 597.2 EU/mL (95% CI: 541.7–658.5) for anti-HPV-18 in subjects seronegative at baseline for the type analyzed. *Post hoc* mathematical modeling predicted a durability ≥50 years for anti-HPV-16 and anti-HPV-18. For the non-vaccine humoral type response, all initially seronegative subjects had seroconverted at M7, with anti-HPV-31 GMT of 2030.5 EU/mL (95% CI: 1766.2–2334.4) and anti-HPV-45 GMT of 2300.8 EU/mL (95% CI: 2036.8–2599.0). At M120, 87.7% and 85.1% remained seropositive for anti-HPV-31 with GMT of 242.9 EU/mL (95% CI: 201.4–293.0) and anti-HPV-45 with GMT of 204.7 EU/mL (95% CI: 170.0–246.6). During the 10-year follow-up, no SAEs or abnormal pregnancy outcomes were causally related to vaccination. Three doses of the AS04-HPV-16/18 vaccine induced high and sustained antibody response against HPV-16,18,31 and 45 in girls aged 10–14 years during the 10-year follow-up, with an acceptable long-term safety profile.

## Introduction

Cervical cancer is one of the leading causes of female cancer worldwide.^1^ Persistent oncogenic human papillomavirus (HPV) infection is necessary for developing cervical cancer.^2^ At least 13 oncogenic types of HPV have been identified; among them, HPV-16 and HPV-18 cumulatively account for approximately 70% of cervical cancer cases.^3^ Other oncogenic types include HPV-45 and HPV-31 that account together for an additional 8.5% of all cervical cancer cases worldwide.^1^

The risk of cervical HPV infection starts soon after sexual debut;^4^ and the risk of HPV infection persists throughout the active sexual life of women.^5^ Therefore, an HPV vaccine that induces a sustained immune response and provides long-term protection is of paramount importance for an HPV vaccination program that targets young girls before sexual debut.^6–8^

The AS04-adjuvanted HPV-16/18 vaccine (AS04-HPV-16/18, *Cervarix*, GSK) has been licensed since 2007 in over 130 countries worldwide. It contains L1 recombinant proteins from HPV-16 and HPV-18, each assembled as virus-like particles and adjuvanted with AS04. The AS04-HPV-16/18 vaccine was shown to be highly efficacious in protecting against HPV-16 and HPV-18 infections and related pre-cancerous cervical lesions as well as protective against some non-vaccine oncogenic HPV types in adolescents and young adults aged 15–25 years.^9–14^ In this age population, the vaccine-induced immune response was shown to persist at a substantial level up to at least 10 years.^15–17^

We conducted a large Phase III study to assess the safety and immunogenicity of HPV-16/18 AS04-adjuvanted vaccine in adolescent girls aged 10–14 years (NCT00196924), the follow-up of which lasted for 10 years (NCT00316706 and NCT00877877). Previous results showed that the vaccine was highly immunogenic and well tolerated, and the high immune response persisted up to 6 years after primary vaccination.^18–20^ Here we report the persistence of antibody response against vaccine types HPV-16 and -18 as well as non-vaccine types HPV-31 and -45 up to 10 years after primary vaccination in this age group. In addition, statistical models were implemented to predict the long-term persistence of HPV-16/18 antibodies after primary vaccination at 10–14 years of age.

## Results

### Subject disposition and demographics

In the primary study (NCT00196924),^19^ 1,035 adolescent girls received at least one dose of the HPV-16/18 AS04-adjuvanted vaccine. Of these, a total of 625 (60.4%) participants who were included in the immunogenicity subset and enrolled from 26 centers in Taiwan, Germany, Honduras, Panama and Colombia participated in the 4-year follow-up (NCT00316706).^20^ All 625 subjects were invited, and of these, 557 (53.8%) subjects participated in this extended follow-up study (NCT00877877) and were included in the total vaccinated cohort (TVC). Four hundred and ninety-five (47.8%) participants completed the study up to 120 months. The mean age of these 495 subjects at Month 120 was 22.0 years (standard deviation [SD]: ±1.4); 46.1% were Hispanic, 32.9% were Caucasian/White and 20.0% were of Asian origin. The demographic characteristics of the participants who completed the 120-month follow-up study were similar to those of the participants enrolled in the primary study.^19^

**Figure 1.**
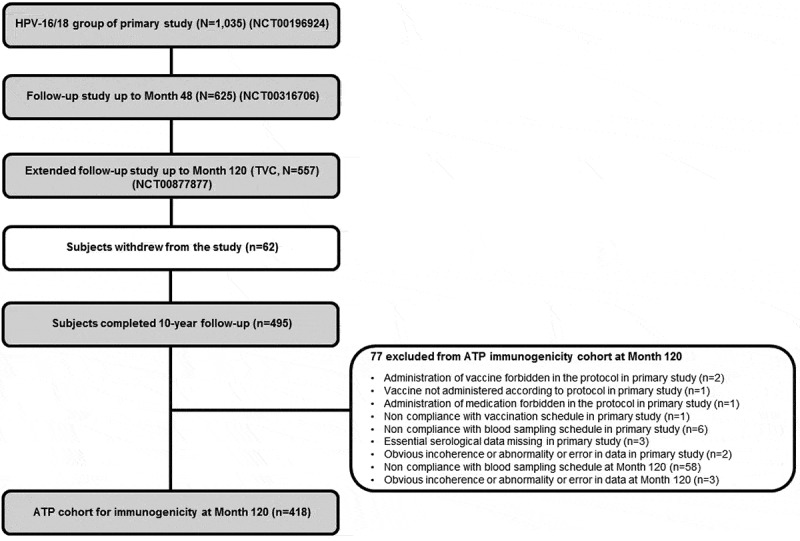
Study design flow chart with the disposition of subjects from the primary study to follow-up studies. ATP, according-to-protocol; HPV: Human Papillomavirus; n: number of seropositive subjects; N: number of tested subjects; TVC, total vaccinated cohort.

At Month 120, 418 subjects were included in the according-to-protocol (ATP) cohort for immunogenicity and 77 subjects were eliminated for ATP analysis for various reasons (Figure 1).

For the *post hoc* analysis on the humoral response against the non-vaccine type HPV-31 and HPV-45, 150 subjects with residual serum volume greater than 300 µL were randomly selected from the ATP cohort.

### Serum antibody response against HPV-16 and HPV-18

Among the 418 subjects who were included in the ATP immunogenicity cohort, anti-HPV-16 antibody data and anti-HPV-18 antibody data were available for 416 and 415 subjects, respectively. At the baseline, 393 (94.5%) out of the 416 subjects were seronegative for anti-HPV-16 antibodies and 395 (95.2%) out of the 415 subjects were seronegative for anti-HPV-18 antibodies. At Month 120 post vaccination, all subjects who were included in the ATP immunogenicity cohort were still seropositive for anti-HPV-16 and anti-HPV-18 antibodies. The geometric mean titer (GMT) values at Month 120 of initially seronegative subjects were 1,589.9 enzyme-linked immunosorbent assay unit [EU]/mL (95% confidence interval [CI]: 1,459.8–1,731.6) for HPV-16 and 597.2 EU/mL (95% CI: 541.7–658.5) for HPV-18. The GMT values at Month 120 of initially seropositive subjects were 1,950.1 EU/mL (95% CI: 1,416.9–2,683.8) for HPV-16 and 866.0 EU/mL (95% CI: 533.4–1,406.1) for HPV-18. The Month 120 antibody GMTs between the initially seronegative and seropositive subjects were not significantly different as the 95% CIs were largely overlapping for both anti-HPV-16 and -18.

**Figure 2.**
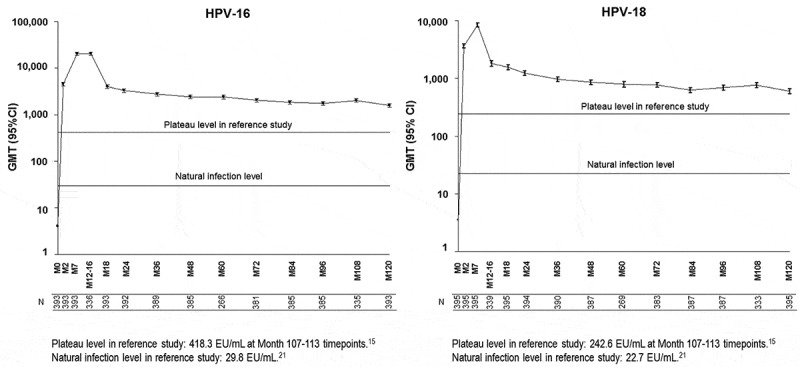
GMTs for anti-HPV-16 and anti-HPV-18 antibodies in initially seronegative subjects (Month 120 ATP immunogenicity cohort). CI: 95% Confidence interval; ELISA: Enzyme-linked immunosorbent assay; EU: ELISA units; GMT: Geometric Mean Titer; HPV: Human papillomavirus; M: Month.

The HPV-16 and HPV-18 antibody titers at Month 120 were 53.4-fold [95% CI: 47.4–60.1] and 26.3-fold [95% CI: 23.6–29.4] higher, respectively, than those observed after natural infection in subjects 15–25 years of age (NCT00122681) (Figure 2).^21^ The HPV-16 and HPV-18 antibody titers at Month 120 were 3.8-fold [95% CI: 3.1–4.6] and 2.5-fold [95% CI: 2.0–3.1] higher, respectively, than those measured 9.4 years after vaccination in subjects vaccinated at the ages of 15–25 years (NCT00518336)(Figure 2).^15^

### Serum antibody response against HPV-31 and HPV-45

**Figure 3.**
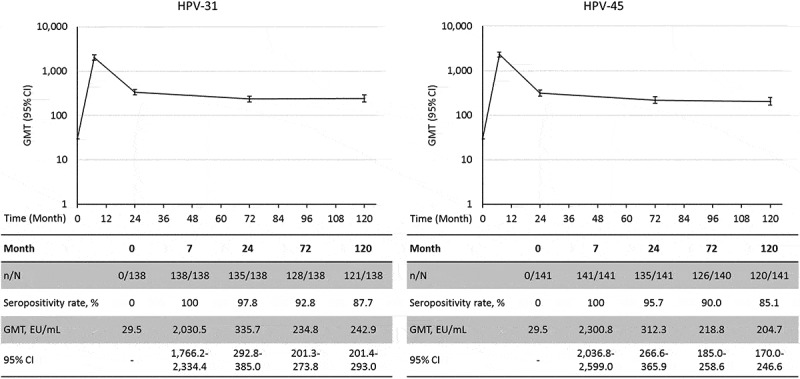
GMTs for anti-HPV-31 and anti-HPV-45 antibodies in initially seronegative subjects (Month 120 ATP immunogenicity cohort). CI: 95% Confidence interval; ELISA: Enzyme-linked immunosorbent assay; EU: ELISA units; GMT: Geometric Mean Titer; HPV: Human papillomavirus; n: number of seropositive subjects; N: number of tested subjects.

For the *post hoc* analysis on the serum antibody response against the non-vaccine type HPV-31 and HPV-45, 150 subjects with residual serum volume greater than 300 µL were randomly selected from the ATP cohort. Among them, 138 (92%) were seronegative for anti-HPV-31 antibodies and 141 (94%) were seronegative for anti-HPV-45 antibodies at the baseline. All of those who were seronegative for the type analyzed at baseline had seroconverted for HPV-31 and HPV-45 antibodies at Month 7; at Month 120, 87.7% and 85.1% remained seropositive for HPV-31 and HPV-45 antibodies, respectively (Figure 3). The GMT for HPV-31 antibodies peaked at Month 7 [2,030.5 EU/mL; 95% CI: 1,766.2–2,334.4] and decreased until Month 24 after which a plateau was observed until Month 120 [242.9 EU/mL; 95% CI: 201.4–293.0]. Similarly, the GMT for HPV-45 antibodies peaked at Month 7 [2,300.8 EU/mL; 95% CI: 2,036.8–2,599.0] and decreased to a plateau level sustained from Month 24 until Month 120 [204.7 EU/mL; 95% CI: 170.0–246.6]. Among the 12 initially anti-HPV-31 seropositive subjects, GMTs were 2,664.9 EU/mL (95% CI: 1,604.5–4,426.0) at Month 7 and 387.6 EU/mL (95% CI: 200.9–748.0) at Month 120. Among the nine initially anti-HPV-45 seropositive subjects, GMTs were 3,351.4 EU/mL (95% CI: 2,225.1–5,047.8) at Month 7 and 362.0 EU/mL (95% CI: 194.7–672.8) at Month 120. The antibody GMTs at the different time points between the initially seronegative and seropositive subjects were not significantly different as the 95% CIs were largely overlapping for both anti-HPV-31 and -45.

### Predicted long-term persistence of serum antibody responses against HPV-16 and HPV-18

**Figure 4.**
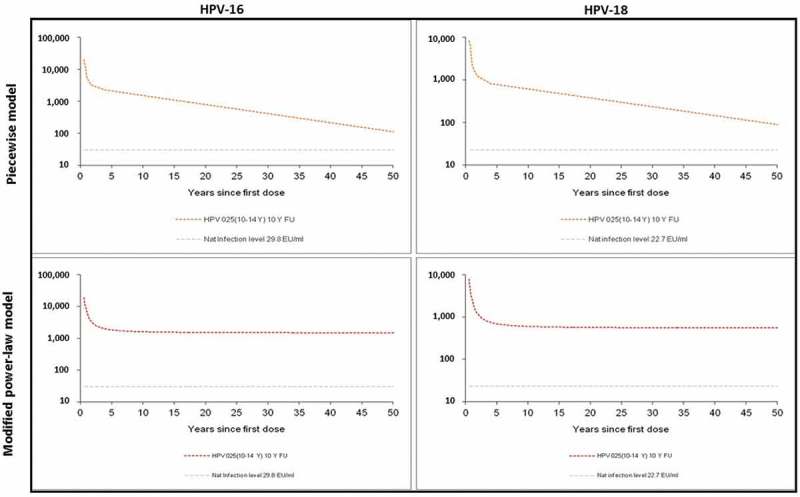
Predicted antibody responses against HPV-16 and HPV-18 over 50 years using Piecewise model and modified power-law model. ELISA: Enzyme-linked immunosorbent assay; EU: ELISA unit; FU: Follow-up; HPV: Human Papillomavirus; Y: Year.

Statistical modeling included the results for subjects who received three doses of the vaccine and for whom data were available at each time point until Year 10 to predict the durability of HPV-16 and HPV-18 antibody response. Figure 4 shows the predicted kinetics of anti-HPV-16 and anti-HPV-18 antibody responses over 50 years after the first dose of vaccination. The modified power-law model predicted a stable antibody response. The piecewise model predicted a slow decay of antibody response which, however, remained well above natural infection levels over 50 years after vaccination (Figure 4). Considering the interpersonal variability in terms of vaccine responses, additional model-based estimations were performed to predict the durability, ensuring that 95% of the subjects would still have anti-HPV-16 and -18 antibody levels above those induced by natural infection. The piecewise model predicted the durability of antibody levels above natural infection of 70.1 years for anti-HPV-16 and of 78.8 years for anti-HPV-18. The modified power-law model predicted lifelong durability for both anti-HPV-16 and anti-HPV-18 (Table 1).

**Table 1.** Predicted duration ensuring 95% of women have HPV-16 and -18 antibody titers above antibody titers after natural infection. ELISA: enzyme-linked immunosorbent assay; EU: ELISA unit; GMT: geometric mean titer; HPV: human papillomavirus.

Antigen	Antibody level at natural infection (EU/mL)	Model	Population GMTs at 50 years post vaccination (EU/mL)	Predicted time for antibody levels above natural infection in 95% participants (years)
HPV-16	29.8	Piecewise	111.2	70.1
		Modified power-law	1,493.9	Always
HPV-18	22.7	Piecewise	90.3	78.8
		Modified power-law	555.4	Always

### Safety

During the follow-up period from Month 0 to Month 120, a total of 99 subjects (17.8%) reported 155 serious adverse events (SAEs). One case of SAE (abdominal pain) was reported 4 days after the first dose of vaccination. Four cases of SAE were reported between the second and third dose of vaccination: injury with time-to-onset (TTO) of 7 days, gunshot wound (TTO: 41 days), bacterial pneumonia (TTO: 42 days) and abdominal pain (TTO: 54 days). Five cases of SAE were reported within 6 months after the third dose of vaccination: dehydration, gastroenteritis and upper respiratory tract infection developed in one subject (TTO: 15 days), herpangina (TTO: 16 days) and syncope (TTO: 26 days). One hundred forty-five cases of SAE were reported during the follow-up period of Years 2–10; the most commonly reported SAE during this period was appendicitis (n = 9). None of these 155 SAEs was considered causally related to vaccination and none of them had a fatal outcome. No participants withdrew from the study because of an adverse event (AE)/SAE.

Among the 557 subjects who participated in the 10-year follow-up, a total of 161 pregnancies were reported by 128 subjects. All pregnancies started at least one year after the third dose of HPV-16/18 vaccination. Among these pregnancies, 134 (83.2%) pregnancies resulted in live infants with no congenital anomaly, 2 pregnancies resulted in live infants with congenital anomalies (one with developmental hip dysplasia and ventricular septal defect and one with congenital megaureter, hydronephrosis, and talipes), 1 pregnancy resulted in stillbirth, 11 pregnancies resulted in spontaneous abortions (10 with no congenital anomaly and 1 with skull malformation of the fetus), and 5 pregnancies were terminated electively (4 with no congenital anomaly and 1 due to suspected renal agenesis and intrauterine growth retardation of the fetus), 2 were ectopic pregnancies, and 6 pregnancies were either ongoing or lost to follow-up at the end of the study. None of the abnormal pregnancy outcomes including the congenital anomalies was considered causally related to the vaccination by the investigators.

**Figure 5.**
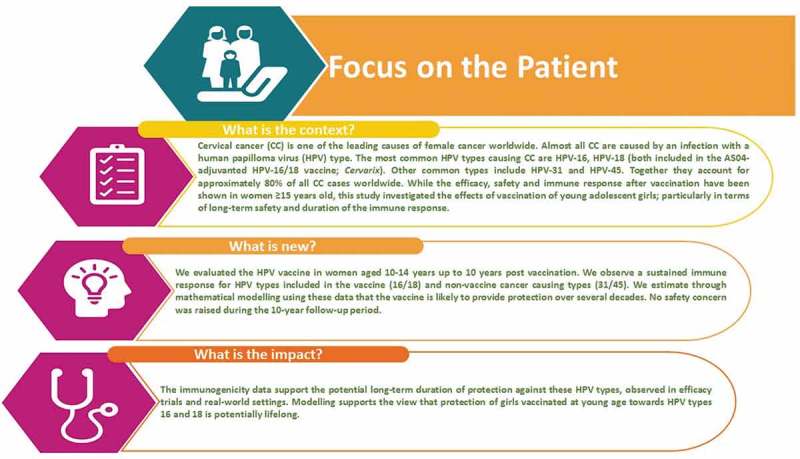
Focus on the patient.

Figure 5 elaborates on the clinical relevance of the trial in a form that could be shared with patients by health-care professionals.

## Discussion

Because of the prophylactic nature of HPV vaccines, the primary target populations in many national immunization programs are young adolescent girls. To our knowledge, this is the longest follow-up study in girls aged 10–14 years who received three doses of the AS04-HPV-16/18 vaccine.

Ten years after the initial vaccination, all subjects remained seropositive for anti-HPV-16 and anti-HPV-18 antibodies. The kinetic profiles of anti-HPV-16 and anti-HPV-18 antibody titer, as well as the non-vaccine type HPV-31 and HPV-45 antibody titer, were similar: a peak response in antibody GMTs one month after administration of Dose 3 (i.e. Month 7), followed by a decline from Month 18 to Month 24 and thereafter reaching a plateau level till Month 120. Such antibody kinetics were also observed in other age populations.^10,16,22,23^

The anti-HPV-16 and anti-HPV-18 antibody plateau levels in young girls aged 10–14 years at first vaccination according to a 3-dose schedule remained several fold higher than the plateau levels observed in young women aged 15–25 years, the age population in which prophylactic vaccine efficacy was demonstrated against high-grade lesions.^11,15^ There is no established immune correlate of protection for prophylactic HPV vaccines. However, the sustained high antibody levels are particularly relevant in supporting the long-lasting protection provided by HPV-vaccination that mainly targets young adolescents, with the intent of rendering protection at the time of exposure.^24^

Aside from its high efficacy against persistent HPV-16/18 infections, results from AS04-HPV-16/18 vaccine trials showed a protective effect of the vaccine against non-vaccine types.^14,25^ In a Phase III clinical trial setting, efficacy against non-vaccine types HPV-31 and HPV-45 has been observed up to 7 years in women vaccinated at 26 years of age and above.^26^ In two studies investigating the 2-dose schedule, cross-reactivity of the vaccine against HPV-31/45 was found similar after 2 doses (girls aged 9–14 years) or 3 doses (young women aged 15–25 years) and lasted for at least 5 years.^27^ Recently, results from observational studies in Scotland and in the Netherlands reported vaccine effectiveness of the AS04-HPV-16/18 vaccine against HPV-31/33/45 and HPV-31/35/45/52, respectively.^28,29^ The sustained immune response that we observed in this study against HPV-31/45 is aligned with these real-life data that are of crucial importance to assess the potential health impact of AS04-HPV16/18 vaccination programs.

Results from a vaccine trial conducted in North America to assess the immune memory in women who received three doses of the AS04-HPV-16/18 vaccine 7 years earlier showed an increase in the antibody titers of vaccine types (HPV-16/18) as well as non-vaccine types (HPV-31/45) following administration of the challenge dose.^30^ These observations evidence the immune memory against HPV-16/18/31/45 induced by the AS04-HPV-16/18 vaccination.

Long-lasting protection is crucial as women are at risk of HPV infection throughout their active sexual life. Conducting a follow-up for several decades in a clinical trial setting is operationally unfeasible as patient retention in clinical trials cannot be maintained over such a long period due to reasons including subjects’ relocation and loss of interest. Therefore, we adopted mathematic models to predict the long-term persistence of anti-HPV-16 and anti-HPV-18 antibodies. Previously, we had performed prediction on antibody persistence using the observed 6-year data, with modified power-law model and piecewise model.^18^ At the end of the 10-year follow-up, we found that the observed antibody titers were lower than those predicted by the modified power-law model but higher than those predicted by the piecewise model (data not shown). In this report, we introduced a fourth break point (i.e. Month 48) in the piecewise model, as such the predicting period could be extended to 50 years. To validate this piecewise modeling with four break points (i.e. Months 7, 12, 21, 48), we performed the model prediction of 10-year antibody kinetics using the 6-year observed data. The predicted antibody kinetic curves fitted well with the observed 10-year antibody kinetic curves (see supplementary section), suggesting that this piecewise modeling with four break points (i.e. Months 7, 12, 21, 48) provided a good prediction. With this model, it has been predicted that the serum anti-HPV-16 and anti-HPV-18 antibody response induced by HPV-16/18 AS04-adjuvanted vaccine remains substantially above the antibody response induced by the natural infection among the young adolescent population for at least 50 years. The durability of the anti-HPV-16 and anti-HPV-18 antibodies among the majority of this population would last for approximately 70 years. These predictions are informative until long-term observational data are available. Due to the lack of serum samples at important time points Month 12 and Month 48, necessary for building the model, the prediction of non-vaccine type anti-31 and anti-45 antibody response beyond 10 years was not performed.

The vaccine was generally well tolerated with a clinically acceptable safety profile during the 10 years of follow-up. There were neither SAEs reported to be causally related to the vaccination nor fatalities. During the entire ten-year follow-up, no adverse pregnancy outcome was considered as related to the vaccine by the investigators. Although a direct comparison was difficult, rates of pregnancy outcomes observed in this trial were found to be close to the reported rates available in the literature.^31–35^ These observations are in line with results from pooled analyses and post-marketing studies conducted so far to assess pregnancy outcomes after exposure to AS04-HPV-16/18 vaccine.^36–39^

The study has some limitations. Notably, the study assessed the 3-dose vaccination schedule, while current vaccination programs in most countries recommended the 2-dose schedule in adolescent girls aged younger than 15 years. The reduced dose schedule from 3-dose to 2-dose for the young adolescent population was based on the evidence that the 2-dose regimens in young adolescent girls showed an equivalent immune response to the 3-dose regimen in 15–25 years old females in terms of magnitude and durability of the immune response.^40,41^ Studies following vaccinated individuals for 6–12 years suggested that 3-dose HPV vaccines offer long-term protection against HPV infection and HPV-related diseases.^42–44^ However, whether the duration of protection can cover the entire sexually active life expectancy and whether a boost dose is required remained to be studied. As a matter of fact, a previous study demonstrated that the 3-dose regimens in young adolescent girls could induce an immune response at least two folds higher than those in 15–25 years-old females,^22^ which was likely to be translated into a longer duration of protection. This study showed that the high immune response induced by the 3-dose vaccine regimen in young adolescent population was sustainable and potentially can last for approximately 7 decades. Another limitation is related to the detection of rare AEs which is limited by the sample size. There was no control group for the follow-up phase as only the subjects who received the HPV-16/18 AS04-adjuvanted vaccine were enrolled. Finally, efficacy endpoints and cervicovaginal secretions as immune response endpoints were not evaluated, which could have provided relevant information.

In conclusion, this study showed that 3 doses of the HPV-16/18 AS04-adjuvanted vaccine induced high and sustained levels of antibodies against HPV-16, 18, 31 and 45 with an acceptable safety profile for at least 10 years after initial vaccination in young adolescent girls aged 10–14 years. The novel prediction of immunogenicity persistence for the vaccine against HPV-16 and -18 for at least 50 years is of significance. Even though no correlate of protection was established, such level of antibody titers may be indicative of protection against HPV-16/18 for a woman up to her sixties and potentially lifelong, should she have received the HPV vaccine in her early teens, suggesting that a subsequent booster dose may not be required. These findings support the feasibility of organized vaccination programs in young adolescents prior to their sexual debut and first HPV exposure.

## Methods

### Study design and subjects

The primary randomized controlled, observer-blinded study (NCT00196924) included female subjects aged 10–14 years at the time of first vaccination who received three doses of the AS04-adjuvanted HPV-16/18 vaccine (*Cervarix*, GSK) or a hepatitis A virus control vaccine (*Havrix*, GSK) at Months 0,1 and 6. Dosage and route of administration of both vaccines can be found in the primary publication.^19^ The subjects who received 3 doses of the HPV-16/18 vaccine during the initial study (from June 2004 to August 2005) were included in the immunogenicity subset (from selected study centers in Taiwan, Germany, Honduras, Panama, and Colombia) and had participated in the 4-year follow-up (NCT00316706, from April 2008 to January 2009) were invited to participate in the current long-term follow-up study (NCT00877877) for up to 10 years after initial vaccination (from May 2009 to January 2015).

Subjects were excluded from the study if they were concurrently participating in another clinical study and had been exposed to an investigational medicinal product, or were receiving additional dose(s) of an HPV vaccine during the follow-up period.

The guidelines of the Declaration of Helsinki, the International Conference on Harmonization-Good Clinical Practices were followed while conducting the study. The study was in accordance with the regulatory requirements of all countries in which the study was conducted, and all subjects provided written informed consents before participating. For subjects below the legal age of consent, written informed consent was obtained from parents or legal representatives as well as assent from the subjects themselves. Subjects were re-consented when having reached the legal age of consent. The study protocol and amendment, as well as informed consent and assent, were reviewed and approved by the independent ethics committee or institutional review board of each study center. Anonymized individual participant data and study documents can be requested for further research from www.clinicalstudydatarequest.com.

### Immunogenicity assessment

In this 10-year follow-up, blood samples were collected at baseline, Months 7, 12, 18, 24, 36, 48, 60, 72, 84, 96, 108, and 120. Antibody titers for anti-HPV-16 and anti-HPV-18 were measured in serum using enzyme-linked immunosorbent assay (ELISA) with type-specific L1 virus-like particles as coating antigen that was produced on a Hi-5 cell line using a baculovirus expression vector system.^45^ Seropositivity was defined as antibody titers ≥8 EU/mL for anti-HPV-16 or ≥7 EU/mL for anti-HPV-18 between Year 0 and Year 7. From Year 8 onwards, the lower limit of quantification of the assays was reviewed, and a new seropositivity cut-off defined as antibody titers ≥19 EU/mL or ≥18 EU/mL for anti-HPV-16 and anti-HPV-18, respectively, was used.^26^ These changes in the assay had no impact on the overall analysis since the antibody titers were higher than both the old and new cut-off values at all time points for all subjects evaluated.

*A post hoc* analysis was performed to measure anti-HPV-31 and anti-HPV-45 antibody titers in serum at baseline, at Months 7, 24, 72, and 120 by ELISA using in-house type-specific L1 virus-like particles produced from baculovirus expression system.^46^ Seropositivity was defined as antibody titers ≥59 EU/mL for both anti-HPV-31 and for anti-HPV-45.

### Statistical analysis and modeling

Statistical analyses were performed using the Statistical Analysis Systems (SAS) version 9.2. Safety analyses were conducted on the TVC, which included all participants who had received 3 doses of the HPV-16/18 AS04-adjuvanted vaccine in the primary study NCT00196924 and whose data were available for at least one study visit in NCT00196924, NCT00316706, and NCT00877877.

The primary immunogenicity analyses were performed on the ATP cohorts for immunogenicity at different time-points that included subjects who met all eligibility criteria for enrollment without violation of the protocols during the follow-up and for whom results were available. A supplementary immunogenicity analysis was performed on the TVC. Seropositivity rates and GMTs were calculated with exact 95% CI for HPV type-specific antibodies. GMT calculations were performed by taking the anti-log of the mean of the log titer transformations. The primary endpoint was the assessment of serum anti-HPV-16 and anti-HPV-18 antibody titers and seropositivity rates in the ATP cohort up to Month 120. The secondary endpoint included the descriptive comparison of anti-HPV-16/18 antibody titers detected in young adolescents vaccinated at the ages of 10–14 years in this study with those who were vaccinated at the ages of 15–25 years (NCT00518336),^15^ the population in which the vaccine efficacy had been demonstrated, as well as the descriptive comparison with the antibody levels elicited in subjects having mounted a detectable immune response after natural infection (NCT00122681).^21^

A *post hoc* analysis assessed the humoral response against the non-vaccine type HPV-31 and HPV-45, i.e. serum anti-HPV-31 and anti-HPV-45 antibody titers and seropositivity rates in a subset of subjects from the HPV group at baseline, at Months 7, 24, 72, and 120. Timepoints were selected to allow building a kinetic profile of the immune response. Samples with available volume greater than 300 µL from subjects having agreed to the future use of their samples were randomly selected to account for all participating countries.

*Post hoc* mathematical modeling evaluated the durability of vaccine-induced immune response via two different mixed-effects models: piecewise and modified power-law. The piecewise model used a linear function on the logarithmic scale in which the data were fitted on four non-overlapping time intervals (with break points at Months 7, 12, 21, and 48) that corresponded to the observed decay of humoral antibodies. The previous modeling with 6-year data included three break points (Months 7, 12, and 21) in piecewise modeling that predicted population antibody responses above natural infection antibody level up to approximately 20 years.^18^ As our current results provided data for up to 10 years, one more piece or break point was defined for the piecewise model considering the duration for up to 50 years. The selection of this additional break point, i.e. Month 48, was based on the Akaike Information Criteria for the whole model (i.e. all break points).^47^ The modified power-law method that estimated the antibody decay over time after vaccination was based on the dynamic populations of B-cells (activated and memory B-cells) that account for the long-term persistence and antibody plateau. The piecewise and modified power-law models were fitted using MIXED and NLMIXED statistical analyses system procedures, respectively.

### Safety assessment

SAEs reported during the entire ten-year follow-up period (Month 0 to Month 120) were recorded. Pregnancies and pregnancy outcomes were recorded during the ten-year follow-up period.

## Supplementary Material

Supplemental Material

## Figures and Tables

**Table:** 

AE	Adverse event
SAE	Serious adverse event
HPV	Human papillomavirus
AS04	Adjuvant System containing 3-*O*-desacyl-4-monophosphoryl lipid A (MPL) adsorbed on aluminum salt
GMT	Geometric mean antibody titer
CI	Confidence interval
SD	Standard deviation
TVC	Total vaccinated cohort
ATP	According-to-protocol
ELISA	Enzyme-linked immunosorbent assay
TTO	time-to-onset
EU	ELISA unit
VLP	virus-like particle

## Trademark statement

*Cervarix* and *Havrix* are trademarks owned by or licensed to the GSK group of companies.
