# Supplementary material for: A ten-year study of immunogenicity and safety of the AS04-HPV-16/18 vaccine in adolescent girls aged 10-14 years
Source: Hum Vaccin Immunother. 2019 Jul 17;15(7-8):1970–9. doi: 10.1080/21645515.2019.1625644 (PMC6746471; doi:10.1080/21645515.2019.1625644)

**SUPPLEMENTARY SECTION**

The long-term persistence of anti-HPV-16 and anti-HPV-18 antibodies was evaluated in the 10-year follow-up study in girls aged 10-14 years for the HPV-16/18 AS04-adjuvanted vaccine. Predictive modelling analysis via the Piecewise model on the current 10-year data predicted antibody responses against HPV-16/18 for at least 50 years.

The observed Geometric Mean Titer (GMT) values for both anti-HPV-16 and anti-HPV-18 antibodies were consistently comparable to the predicted GMT values by the Piecewise model at the all time-points in the 10-year duration (See Figure 1). In fact, at 10 years the observed values were higher than the predicted values. This correlation between the observed and predicted GMT values validates the reliability of the piecewise model.

LL: Lower Limit of confidence interval; UL: Upper Limit of confidence interval

**FIGURE 1**

**(A) Predicted and observed HPV-16 IgG kinetics up to 10 Years after Dose 1 in subjects who received all 3 doses of HPV vaccine**

**
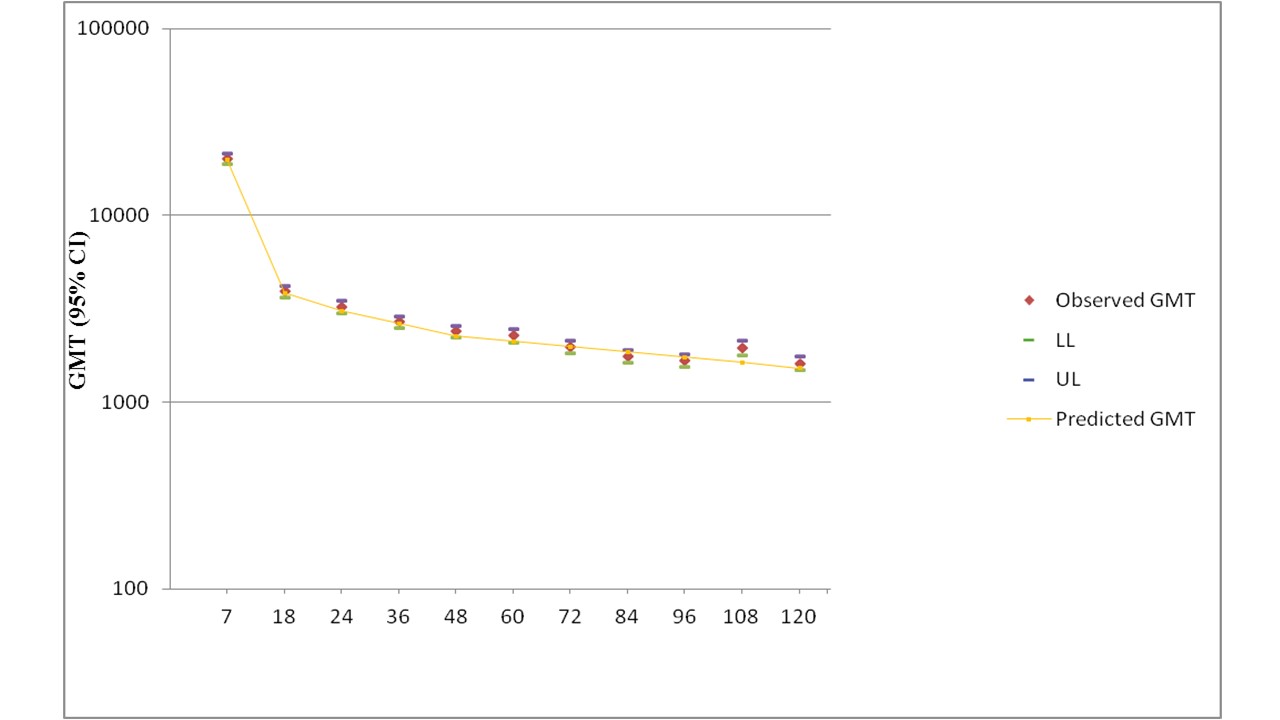
**

**(B) Predicted and observed HPV-18 IgG kinetics up to 10 Years after Dose 1 in subjects who received all 3 doses of HPV vaccine**


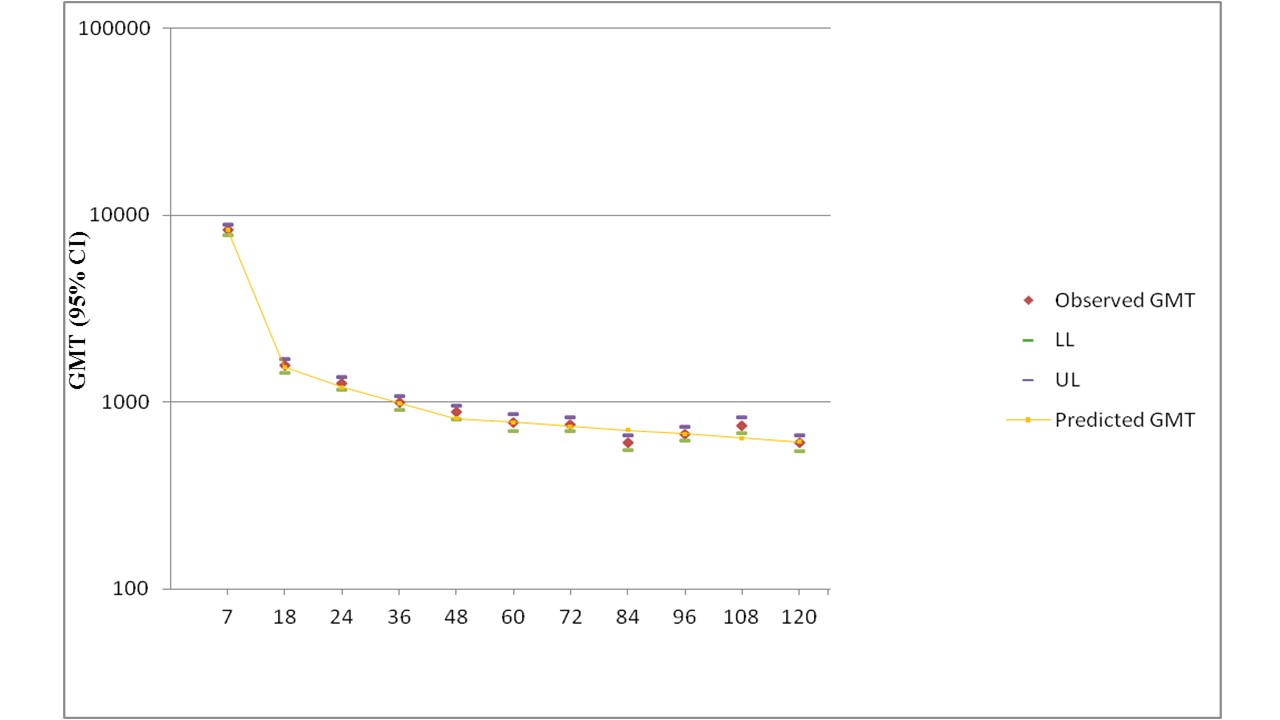

Supplement: Supplemental Material [file khvi-15-7-8-1625644-s001.docx]
